# Pseudogene Lamr1-ps1 Aggravates Early Spatial Learning Memory Deficits in Alzheimer’s Disease Model Mice

**DOI:** 10.1007/s12264-024-01336-6

**Published:** 2025-01-02

**Authors:** Zhuoze Wu, Xiaojie Liu, Yuntai Wang, Zimeng Zeng, Wei Chen, Hao Li

**Affiliations:** 1 Institute of Basic Medicine and Forensic Medicine, North Sichuan Medical College, Nanchong, 637100 China; 2 School of Clinical Medicine, North Sichuan Medical College, Nanchong, 637100 China; 3 Department of Pathophysiology, School of Basic Medicine and Tongji Medical College, Huazhong University of Science and Technology, Wuhan, 430030 China

**Keywords:** Alzheimer’s disease, Lamr1-ps1, Pseudogene, LncRNA, Learning and memory, MiR-29c-3p

## Abstract

Alzheimer’s disease (AD), a neurodegenerative disorder with complex etiologies, manifests through a cascade of pathological changes before clinical symptoms become apparent. Among these early changes, alterations in the expression of non-coding RNAs (ncRNAs) have emerged as pivotal events. In this study, we focused on the aberrant expression of ncRNAs and revealed that Lamr1-ps1, a pseudogene of the laminin receptor, significantly exacerbates early spatial learning and memory deficits in APP/PS1 mice. Through a combination of bioinformatics prediction and experimental validation, we identified the miR-29c/Bace1 pathway as a potential regulatory mechanism by which Lamr1-ps1 influences AD pathology. Importantly, augmenting the miR-29c-3p levels in mice ameliorated memory deficits, underscoring the therapeutic potential of targeting miR-29c-3p in early AD intervention. This study not only provides new insights into the role of pseudogenes in AD but also consolidates a foundational basis for considering miR-29c as a viable therapeutic target, offering a novel avenue for AD research and treatment strategies.

**Supplementary Information:**

The online version contains supplementary material available at 10.1007/s12264-024-01336-6.

## Introduction

Alzheimer’s disease (AD) is a well-known neurodegenerative disease with an insidious onset and a long-term incubation process and characterized by the deposit of extracellular amyloid-β (Aβ) in senile plaques and neurofibrillary tangles (NFTs) caused by tau protein hyperphosphorylation [1, 2]. The incidence of AD increases with age and is aggravated due to the current trend of an aging population [3]. Significant memory loss and cognitive dysfunction occur in the clinical stage of AD, but before that, Aβ deposition and NFTs might have occurred already. Meanwhile, neuroinflammation or impairment of synaptic function could have also been reported in the pre-onset stage of AD [4]. The pathogenesis of AD is multifaceted and complex. In recent years, a number of reports have focused on the mechanisms of action of early neuropathology, such as neuroinflammation. However, there is still a need to deepen the clinical aspects of treatment [5]. Consequently, the exploration of the mechanisms of disease pathology in the progress during AD, especially in the early stage, can help with its diagnosis and treatment.

As a common epigenetic regulatory mechanism, the alteration of noncoding RNAs (ncRNAs) could serve as early events of aberrant gene expression [6]. Long noncoding RNAs (lncRNAs) are defined as being longer than 200 nt. They cannot encode proteins directly due to the lack of open reading frames, but their abundant presence and widespread localization enable them to influence their target proteins in a multitude of biological processes at either the transcription or translation level [7]. In addition, the tissue-specific and conditional expression patterns suggest that lncRNAs provide a theoretical basis as potential biomarkers for clinical targets [8]. Evidence accumulated over decades has shown that lncRNAs ultimately affect the diverse pathophysiological contexts of AD, such as Aβ aggregation, neuronal apoptosis, and oxidative stress [9]. For instance, the lncRNA BACE1-AS, a non-coding antisense RNA of BACE1, can act as a competitive endogenous RNA (ceRNA) to stabilize BACE1, leading to additional Aβ aggregation [10], or to activate autophagy-related gene 5 (ATG5), which induces neuronal damage in AD through autophagy [11]. In terms of neuroapoptosis, some lncRNAs, such as SNHG1 and BDNF-AS are involved by acting as miRNA-adsorbing sponges [12, 13]. These studies reveal the underlying molecular mechanisms of lncRNAs in AD pathology, suggesting that they play important roles in various aspects. However, the occurrence of AD involves long-term dysregulation of the transcript expression profiles. There is a need for an in-depth investigation of the altered expression and molecular mechanisms of lncRNAs spanning the pre-morbid stage to late-onset AD.

In this study, we investigated the alterations of lncRNA expression in the hippocampus of 3-, 6-, and 12-month-old APP/PS1 mice compared to age-matched wild-type (WT) mice. These three age groups of this AD model were chosen to simulate the uninitiated, pre-morbid, and late stages of AD, as cognitive behavioral impairments in these mice have been reported to appear after 6 months and worsen with age [14]. Through the comparisons of differentially-expressed lncRNAs by the three age groups of APP/PS1 mice with their respective counterparts as noted in our previous work [15], lncRNA Lamr1-ps1, a pseudogene of the laminin receptor (Lamr1), was found to be up-regulated in APP/PS1 mice. Pseudogenes were initially regarded as “nonfunctional” genomic elements until the widespread availability of high-throughput sequencing analysis revealed much about pseudogenes [16]. Some pseudogenes have similar biological functions to lncRNAs and may be related to corresponding coding genes [17]. However, there are few reports on the role of pseudogenes in AD. The matched coding genes of Lamr1-ps1, Lamr1, is a multifaceted cellular receptor that plays an essential role in facilitating neurotoxicity in AD [18]. However, the potential molecular mechanism of Lamr-ps1 in AD has not been reported. In the present study, we demonstrate that Lamr1-ps1 promotes the expression of Bace1 as well as aggravating AD-like behaviors in APP/PS1 mice *via* miR-29c-3p. Our findings contribute to the theoretical basis for the potential mechanism of Lamr1-ps1 action in AD.

## Materials and Methods

### Animals

Male C57BL/6 and APP/PS1 mice (RRID: MMRRC_034832-JAX) were bred and reared according to the institutional guidelines for the Care and Use of Laboratory Animals and all animal experiments were approved by the Ethics Committee of North Sichuan Medical College. The mice were kept in groups of 3–5 per cage and housed in a room with a stable ambient temperature of 21 ± 1°C and humidity at 50% ± 5%. The light phase of the 12-h light-dark cycle was from 08:00. All behavioral experiments were conducted at the same time during the light phase.

### Microarray Analysis

The 3-, 6- and 12-month-old APP/PS1 and control mice were sacrificed, and their hippocampi were collected separately, cryoprotected, and stored at -80℃. Then microarray analysis of these tissues was applied using Agilent Mouse lncRNA Microarray V3, which was provided by OE Biotech (Shanghai, China). The procedures were as previously described [15]. Briefly, the total RNA of three replicate mouse hippocampi that were selected randomly from the 6 groups was required to first meet the requirements for quantitative and integrity testing. Subsequently, the processes of sample labeling, microarray hybridization, and cleaning were carried out in accordance with the standard protocol. Through the normalization process as well as the filtering of the quantile algorithm, raw data were presented as a matrix. Differentially-expressed lncRNAs (DElncRs) from any two groups of comparisons were subsequently identified based on the fold change (|Fold Change| >2) and *P* value (*P* value ≤0.05,* t*-test) The online platform (https://www.bioinformatics.com.cn) facilitated the visualization of these DElncRs as clustered heatmaps. In addition, the microarray data featured in this research have been archived in the GEO database under the accession number GSE242902.

### Reverse Transcription and Quantitative Polymerase Chain Reaction (RT-qPCR)

Total RNA from the hippocampus was isolated using TRIzol (15596026, Invitrogen, USA) and then converted to cDNA using a cDNA Synthesis Kit (11141ES60, Yeasen, China), strictly following the manufacturer's guidelines. For reverse transcription, 1 μg of total RNA per sample was incubated with 5× gDNA digester Mix for 2 min at 42℃, then the reaction solution was incubated with 4× Mix to perform a rapid reverse transcription for 15 min at 55℃, and reactions were stopped at 85℃ for 5 min. The cDNA products were only suitable for the subsequent qPCR experiment and needed to be diluted properly. For the qPCR assay, the cDNA products were amplified using the qPCR Mix (11201ES03, Yeasen, China). The Supplementary Tables display all the primers (manufactured by Sangon Biotech, Shanghai, China), and each product amplified by these primers was detected as a single peak in the melting curve. In the experimental setup, reactions occurred in a 10 μL volume comprising 5 μL of 2 × qPCR Mix, 1 μL of a 10 nmol/L primer mixture, and 1 μL of cDNA template which was diluted 1:5 from the RT product. The PCR cycle was as follows: 95℃/3 min, 40 cycles of 95℃/10 s, and 60℃/30 s. qPCR assays were applied to several lncRNAs to validate the microarray analysis results, and the expression levels of mRNAs and lncRNAs were normalized to the internal GAPDH and determined using the 2^−ΔΔCT^ method.

### Morris Water Maze (MWM) Test

The MWM test was applied to examine the spatial learning and memory of mice using the device with WMT-100 software (Tai Meng, Chengdu, China). The water maze is a circular tank (120 cm diameter) filled with opaque water (21 ± 1°C) and a hidden platform (6 cm diameter) submerged 1 cm below the surface. The procedure was as previously described [19]. Briefly, the mice were allowed to acclimate to the testing room for 30 min before the start of training trials. The experiment consisted of two parts: spatial learning trials and probe tests. In the spatial learning trials, the mice were required to locate the unseen platform within 90 s three times per day for 6 straight days. Their escape latency to discover the hidden platform and swimming speed were noted during these sessions. Following a day of rest, the mice were individually subjected to probe tests where they were placed in the water, farthest from the original platform location, and left for 90 s in the absence of a platform. Subsequently, the time each mouse spent in each quadrant was analyzed.

### Novel Object Recognition

The Novel Object Recognition (NOR) test contains three stages: adaptation, training, and testing. Each mouse was placed in the open field box measuring 60 cm × 60 cm × 40 cm for free movement and exploration for 5 min in the first adaptation phase. After each mouse was tested, the odors in the chamber were removed. Then, the training phase was scheduled. Mice were exposed to two identical objects (A and B) which were placed at symmetrical locations in the box and recorded for 5 min. The recognition test was applied on the next day. In this session, one of the old objects (A) was replaced by a novel object (C), and each mouse was allowed to explore the box for 5 min. The time and counts that mice spent exploring the two objects were recorded, and the preference index of each mouse was calculated using the time spent with B and C by the formula C/(B+C) × 100%.

### Fluorescence *In situ* Hybridization (FISH)

After perfusion, the brain was fixed overnight in 4% paraformaldehyde in PBS at 4℃ and subsequently dehydrated in 30% sucrose in PBS (diethylpyrocarbonate-treated) and cut at 20 μm. The RNA probe for Lamr1-ps1 was obtained using a Digoxin (DIG) RNA Labeling Kit (11175025910, Merck, USA), following its instructions. Briefly, the plasmid containing the Lamr1-ps1 sequence and the primers (forward: 5’-AGCCCTCCATACCCTGCAAA-3’, reverse: 5’-TAATACGACTCACTATAGGGTGGGATCAGTCACCACCAGA-3’) used to generate the template PCR product were synthesized by Sangon Biotech (Shanghai, China). Then the DIG-labeled RNA probes were generated by incubation for 2 h with template DNA with the T7 promoter in accordance with the synthesis system of the kit. The purified RNA probes (0.5-2 μg/mL) were used in FISH assays and the experimental procedure was applied using the ISH assay kit (MK1034, Boster, China). It should be pointed out that RNA fragments in brain slices were exposed to 3% pepsin diluted in citric acid at 37°C for 10–120 s. The slices were pre-hybridized after rinsing with PBS and were incubated in pre-hybrid solution for 2–4 h at 37–40°C in the incubator. Then 0.5–2 μg/mL purified RNA probes (diluted in hybrid solution) were incubated with the brain slices overnight at 37–40°C. Then, after washing and sealing, biotinated DIG was added then incubated for 1 h at 37°C, and SABC-FITC was added in turn and incubated for 30 min at 37°C. For co-stained protein immunofluorescence in the same slices, the operations of blocking and primary antibody binding were applied after the incubation with RNA probes was completed.

### Immunohistochemistry

Immunohistochemistry was applied to brain sections following a previously described protocol [20]. Briefly, sections were incubated and blocked in 3% BSA diluted in 0.5% Triton-PBS at room temperature (20–22°C) for 1 h. The sections were incubated overnight at 4°C with one of the following primary antibodies: mouse monoclonal anti-NeuN (1:500, ab104224, Abcam), rabbit polyclonal anti-GFAP (1: 200, ab7260, Abcam), or mouse monoclonal anti-β-Amyloid (1: 200, 800708, Biolegend). For immunohistochemical staining in the next step, Biotin-labeled secondary antibodies (P0612, Beyotime, China) were selected to react with the primary antibodies. Then the slices were examined with a DAB kit (P0202, Beyotime, China). For immunofluorescence staining in the next step, the slices were rinsed with PBS and reacted with conjugate adsorbed Alexa Fluor 546 donkey anti-rabbit or donkey anti-mouse secondary antibodies (Thermo Fisher Scientific, USA). Images were captured under a scanning microscope (Olympus VS120, Japan) or a confocal laser-scanning microscope (Zeiss LSM800, Germany). We randomly selected the brains of three mice from each group for staining and analysis.

### Cell Culture and Treatment

The mouse brain neuroma cell line Neuro-2a (N2a) (CL-0168, Procell, China), and the mouse hippocampal neuronal line, HT22 cells (CL-0697, Procell, China) were cultured in Dulbecco’s modified Eagle’s medium (DMEM) containing 10% fetal bovine serum and 1% penicillin and streptomycin. The cells were maintained in an incubator under 5% CO_2_ at 37°C, and cultured in 6- or 12-well plates for transfection. The scrambled miRNA, miR-29c-3p mimic, and miR-129-1-3p mimic were purchased from Ribobio Corp. (Guangzhou, China), and the Lamr1-ps1 overexpression and knockdown plasmids were designed and generated by ViGene Biosciences (Shandong, China). Aβ_1-42_ oligomers (A9810, Sigma, USA) were treated with hexafluoroethane before addition to the culture medium at final concentrations of 0, 5, and 10 μmol/L, respectively. Three replicates in each treatment were collected 48 h later for follow-up testing.

### Enzyme-Linked Immunosorbent Assay (ELISA) and Western Blot (WB)

The Mouse Aβ_1-42_ ELISA Kit (E-EL-M3010, Elabscience, China) was used to assess the Aβ_1-42_ content in the hippocampus. Tissue samples were weighed and homogenized in PBS containing protease inhibitors [tissue weight (g): PBS (mL) volume = 1:9] on ice. The supernatant after centrifugation (5 min, 5000 g, 4°C) was used for the assay according to the manufacturer’s instructions. Three replicates for each sample were performed and immediately measured at 450 nm.

The total protein of cells or brain tissue with different treatments was extracted by radioimmunoprecipitation assay buffer (P0013, Beyotime Biotechnology, Shanghai, China) containing 100 mg/mL PMSF. After centrifugation at 12000 r/min for 5 min, the supernatant from cell or tissue homogenates was separated, and an appropriate volume of 5× loading buffer was added and boiled for 10 min for WB. The proteins in the samples underwent electrophoresis on a 10% SDS-PAGE gel and were subsequently transferred to nitrocellulose membranes. After blocking with 3% BSA in TBST for 30-60 min, proteins within the membranes were incubated overnight at 4ºC with the following primary antibodies: anti-APP (1: 2000, ab32136, Abcam), anti-Bace1 (1: 1000, BM5148, Boster), anti-Lamr1 (1:1000, 14533-1-AP, Proteintech), and anti-GAPDH (1:20000, 60004-1-Ig, Proteintech). The membranes were washed twice with TBST for 5 min and then incubated with the corresponding secondary antibody at room temperature for 1 h. After washing, the blots were assessed using an ECL kit (P0018, Beyotime, China). Some of the immunoblots in this study were scanned using an Infrared Imaging System (Odyssey, LI-COR).

### Brain Stereotaxic Injection

Mice were anesthetized with isoflurane (2%–5%) and placed in a stereotaxic apparatus. Adeno-associated viruses (AAVs) or miRNA agomir were injected unilaterally or bilaterally into the hippocampus according to the experimental needs. For AAV injections, a total of 2 μL AAV-Lamr1-ps1 or control solution was injected within 20 min using a micro syringe pump as previously described [21]. For miRNA agomir injections, 5 μmol/L agomir powder was first dissolved in 50 μL 0.9% saline, then 2 μL agomir solution was injected as previously reported [22]. After the injection, the needle was left in the brain for 10 min and retracted slowly to avoid leakage. AAV9 at a viral titer of 10^13^ for Lamr1-ps1 overexpression and control were packed by Genechem (Shanghai, China) and agonies for miR-29c-3p were ordered in Ribobio (Guangzhou, China). The MWM test was applied 4 weeks after the virus injection or 3 weeks after the atomic injection.

### Dual-Luciferase Reporter Assay

The predicted target sequences of ~300 bp containing the Lamr1-ps1 binding site to miR-29c-3p or miR-129-1-3p were cloned into multiple cloning sites (between XhoI and NotI) of the psiCHECK2 plasmid. The mutant sequences of the ligated loci were used as their respective controls. The dilute mimics or scrambled control were co-each-transfected into HEK293T cells with the newly constructed psiCHECK2 plasmid. Cells were harvested 48 h after completion of transfection. The cell lysates after centrifugation were used for detection experiments. The dual-luciferase reporter kit (RG027, Beyotime, China) was used to examine the firefly and *Renilla* luciferase activity according to the instructions. Three replicates for each experimental group were harvested and analyzed.

### Statistical Analysis

The data were analyzed using GraphPad Prism software, version 9.0, and are expressed as the mean ± standard error of the mean (SEM). Two groups were compared using a two-tailed Student’s *t*-test, and multiple comparisons were evaluated using two-way ANOVA. The statistical significance of all comparisons was defined as *P <0.05, **P <0.01, or ***P <0.001. The investigators conducting the experiments and collecting data were unaware of the group allocation.

## Result

### Identification and Temporal Expression Patterns of Differentially-expressed LncRNAs in APP/PS1 Mice

**Fig. 1:**
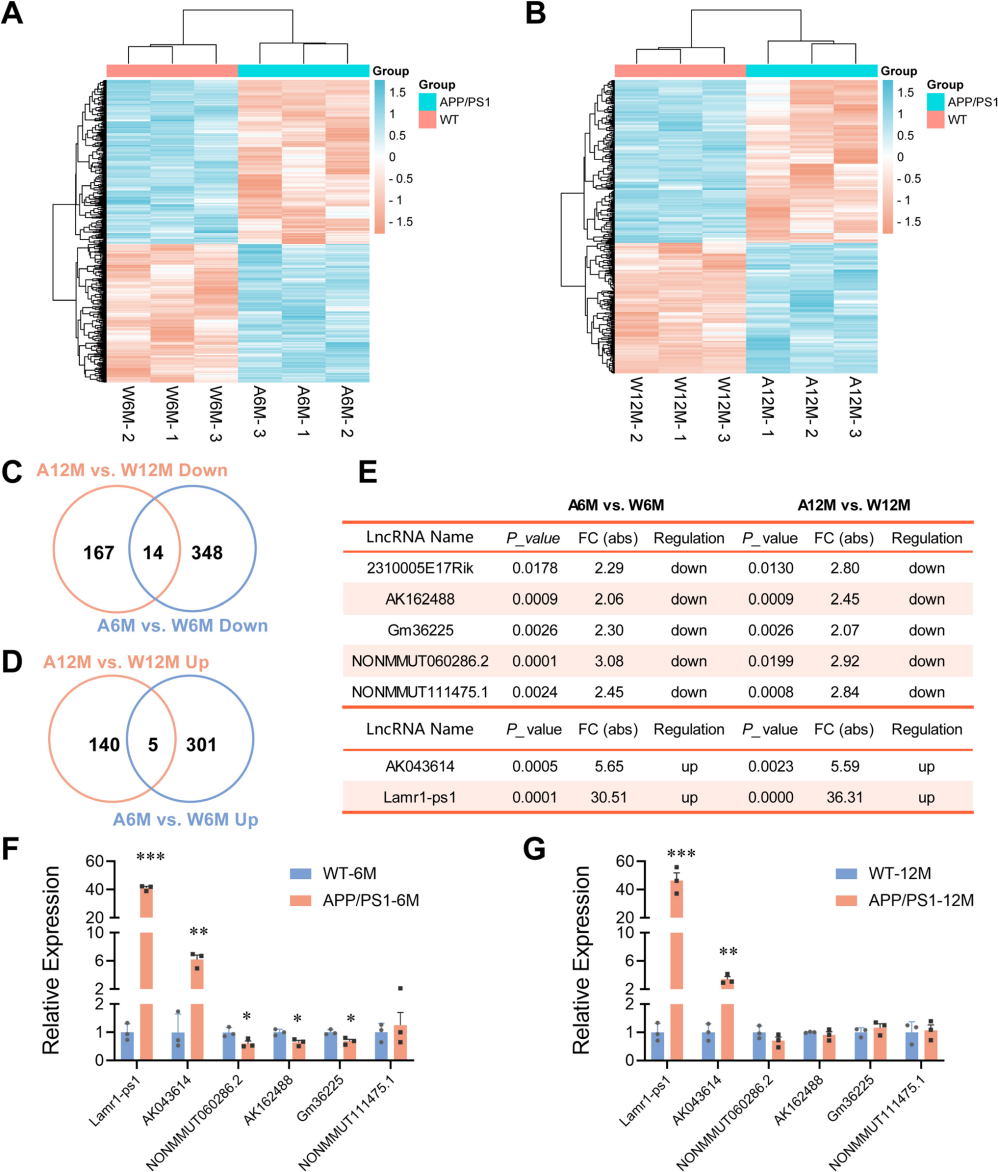
Screening and validation of differentially-expressed lncRNAs in 6- and 12-month-old APP/PS1 mice. **A**, **B** Hierarchical clustering heatmaps based on the normalized expression of DElncRs at 6 (A) or 12 (B) month-old APP/PS1 and WT mice. 6 and 12-month-old APP/PS1 or WT mice were abbreviated as A6M, A12M or W6M, W12M, respectively. **C**, **D** Venn diagrams of down-regulated (C) or up-regulated (D) DElncRs in 6- and 12-month-old APP/PS1 mice compared with age-matched WT mice. **E** The *P*-value and fold change (FC) of 7 selected DElncRs (5 down-regulated and 2 up-regulated genes) in APP/PS1 mice *versus* WT mice. **F**, **G** qPCR validation of the 7 DElncRs in 6- (F) and 12- (G) month-old APP/PS1 mice and age-matched WT mice. In 6-month-old APP/PS1 mice, Lamr1-ps1 (***P <0.001, *t* = 36.26), AK043614 ( **P = 0.002, *t* = 7.070), NONMMUT060286.2 (*P = 0.042, *t* = 2.952), AK162488 (*P = 0.020, *t* = 3.757), and Gm36225 (*P = 0.022, *t* = 3.626) are significantly changed compared with WT mice, while in 12-month-old APP/PS1 mice, only Lamr1-ps1 (***P = 0.001, *t* = 8.421) and AK043614 ( **P = 0.005, *t* = 5.639) are significantly upregulated compared with WT mice. Data are the mean ± SEM (*n* = 3 per group, unpaired two-tailed *t*-test).

In APP/PS1 mice, we found 504, 668, and 326 DElncRs at 3, 6, and 12 months, respectively, compared to control mice of the same age. To analyze changes in transcriptional expression in both the early and late stages of the disease, we focused on the 6- and 12-month-old groups. There were 306 up-regulated as well as 362 down-regulated genes in the 6-month-old mice, while 145 were up-regulated and 181 down-regulated in 12-month-old APP/PS1 mice compared to the respective controls. Hierarchical clustering heatmaps were plotted based on the normalized expression of DElncRs in 6- and 12-month-old APP/PS1 and WT mice, respectively (Fig. 1A, B). Of the down-regulated transcripts, 14 were decreased in both 6- and 12- month-old APP/PS1 mice (Fig. 1C), whereas 5 showed concurrent increases in the up-regulated genes (Fig. 1D). To ensure subsequent validation of these lncRNAs, 7 (5 down- and 2 up-regulated) were screened based on the normalized expression values ≥8 (at least one sample) as listed (Fig. 1E). Their line graphs expressing the trend with age are plotted individually in Fig. S1. Then, we used qPCR to examine the expression of these 7 lncRNAs in 6- and 12-month-old APP/PS1 and their WT counterparts. The results revealed that Lamr1-ps1 is significantly increased in 6- and 12-month-old AD mice (Fig. 1F, G; *P* <0.001).

### Localization and Expression of Lamr1-ps1

**Fig. 2:**
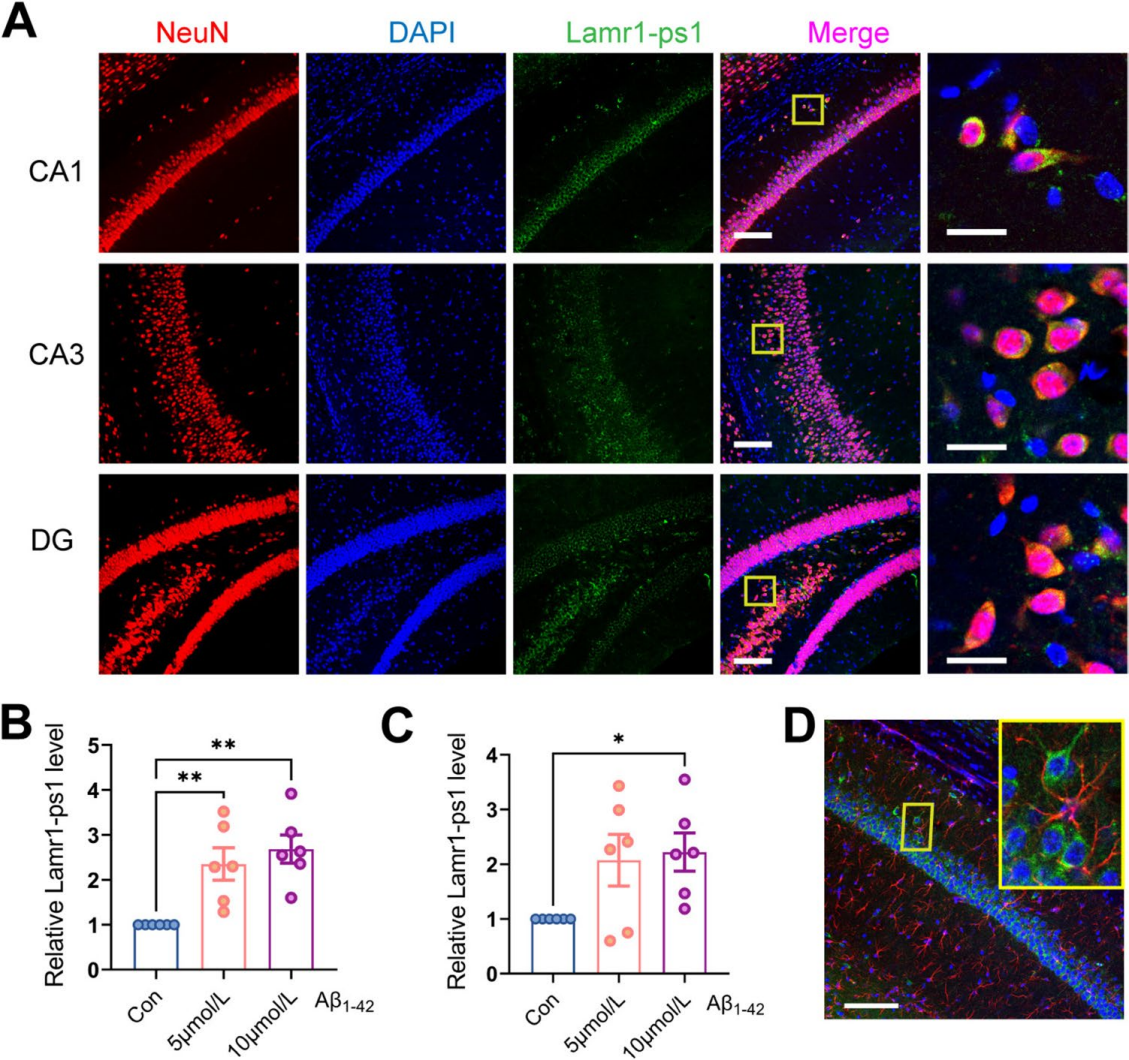
Lamr1-ps1 is mainly expressed in neuronal cells. **A** Representative images showing the expression of Lamr1-ps1 (green) and NeuN (the neuronal marker, red) in the hippocampus of APP/PS1 mice. The stained images in the CA1, CA3, and DG areas are plotted from upper to lower (scale bars, 100 μm), and the merged enlarged images show co-staining of neurons and Lamr1-ps1 (right column, scale bars, 20 μm). **B**, **C** The relative expression of Lamr-ps1 in the N2a (B) and HT22 (C) cells treated with 0, 5, and 10 μmol/L Aβ_1-42_. Data are the mean ± SEM (*n* = 6 per group, in the N2a cell group, *F* = 10.49, Con *vs* 5 μmol/L **P = 0.0065, Con *vs* 10 μmol/L **P = 0.0012; in the HT22 cell group, *F* = 3.850, Con *vs* 10 μmol/L *P = 0.041; one-way ANOVA followed by Bonferroni multiple comparisons). **D** Representative images showing the expression of Lamr1-ps1 (green) and GFAP (a marker of astrocytes, red) in the hippocampus of APP/PS1 mice; the merged images show that astrocytes are not co-stained with Lamr1-ps1 (scale bar, 100 μm).

Lamr1-ps1 is a processed pseudogene of 874 bp on chromosome 2, but investigation of its function is lacking. To extend the over-expression or knockdown experiment, we first determined the cellular localization of Lamr1-ps1 by co-staining Lamr1-ps1 with NeuN (a neuron marker) using a DIG-labeled RNA probe and anti-NeuN antibody in brain slices. Significantly, Lamr1-ps1 was highly co-located with neurons in the hippocampus of AD mice (Fig. 2A). Then we established the AD model of the cell by Aβ_1-42_ infection and assessed how the Lamr1-ps1 level changed in N2a (a mouse brain neuroma cell line) and HT22 cells (a mouse hippocampal neuronal line) treated with different concentrations of Aβ_1-42_. The results showed that the expression of Lamr1-ps1 was significantly increased by Aβ_1-42_ at 10 μmol/L in both N2a and HT22 cells (Fig. 2B, C, *P* = 0.0012 and *P* = 0.041, respectively). This finding suggested that Lamr1-ps1 expression is also elevated in AD neuronal cell models. Although the co-localization of Lamr1-ps1 with neurons was significant, we also co-labeled Lamr1-ps1 with other cells in the brain such as astrocytes, and it was evident that there was no significant overlap between astrocytes and Lamr1-ps1 (Fig. 2D, S2). These results showed that Larm1-ps1 is primarily located in the cytoplasm of neuronal cells, in which it is highly expressed after Aβ treatment.

### Lamr1-ps1 Enhances the Expression of Bace1

**Fig. 3:**
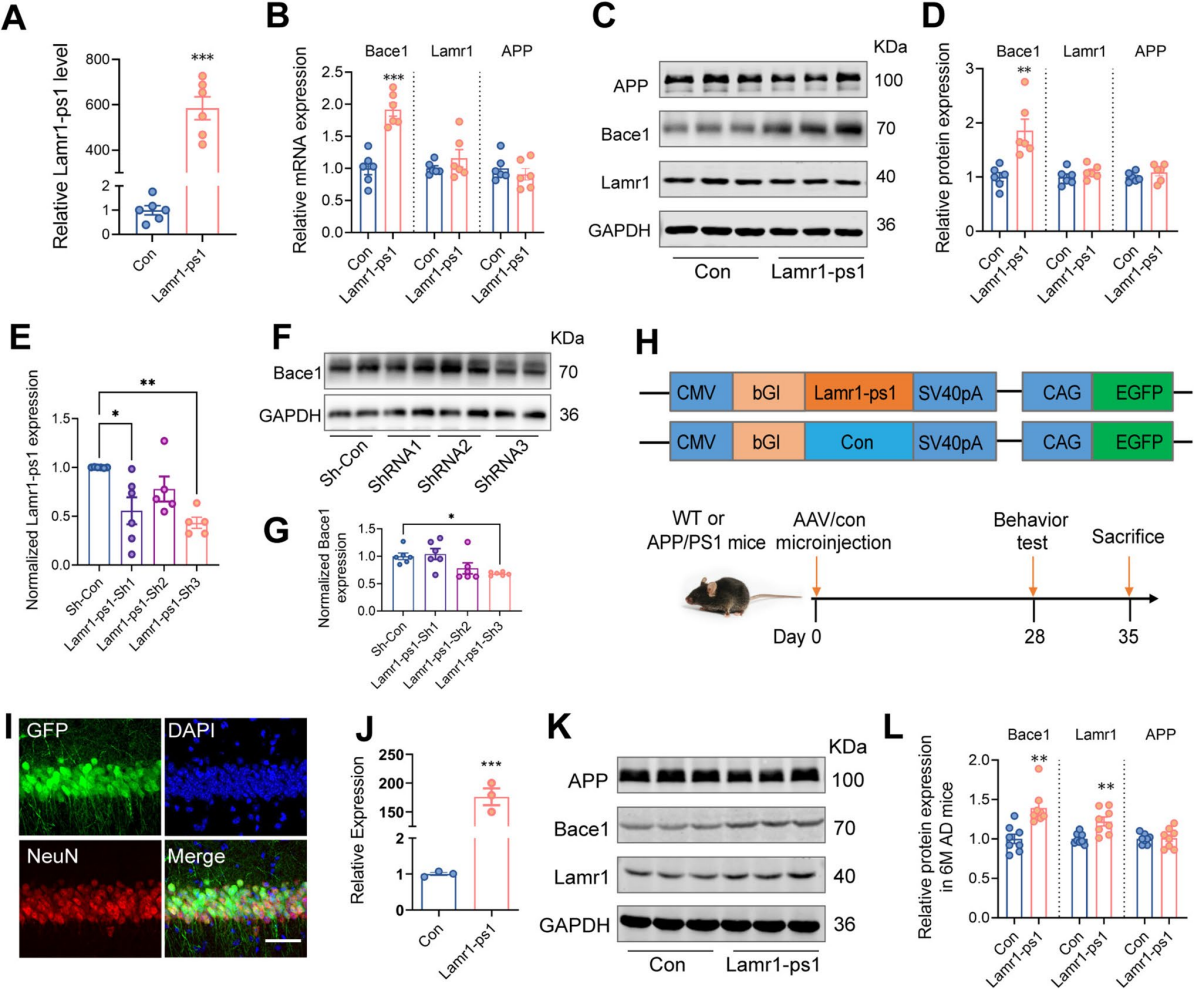
Lamr1-ps1 enhances the expression of Bace1. **A** qPCR validation of Lamr1-ps1 after Lamr-ps1 plasmid is over-expressed in N2a cells. Data are the mean ± SEM (*n* = 6 per group, ***P <0.001, *t* = 11.60, unpaired two-tailed *t*-test). **B** The relative mRNA expression of Bace1, Lamr1, and APP after Lamr-ps1 is over-expressed in N2a cells. Data are the mean ± SEM (*n* = 6 per group (***P <0.001, *t* = 6.423, only Bace1 is upregulated in the Lamr1-ps1-treated group, unpaired two-tailed *t*-test). **C**, **D** Lysates of N2a cells were prepared and blotted with anti-APP, Bace1, Lamr1, and GAPDH, as indicated (C). Relative protein expression of APP, Bace1, and Lamr1 defined by normalizing their respective band intensities to GAPDH (D) (*n* = 6 per group, (**P = 0.0034, *t* = 3.821, unpaired two-tailed *t*-test; Bace1 is upregulated in the Lamr1-ps1-treated group compared to control). **E** The mRNA expression of Lamr1-ps1 in N2a cells treated with ShRNA or control. Data are the mean ± SEM (*n* = 5 per group, Sh-Con *vs* ShRNA1 *P = .0106, Sh-Con *vs* ShRNA3 **P = 0.0022, *F* = 4.506, one-way ANOVA followed by Bonferroni multiple comparisons). **F**, **G** Representative bands (F) of western bolts for anti-Bace1 in N2a cell lysates treated with ShRNA and control plasmids. Relative protein expression of Bace1 (G) in each group. Data are the mean ± SEM (*n* = 6 per group, Sh-Con *vs* ShRNA3 *P = 0.021, *F* = 1.58, one-way ANOVA followed by Bonferroni multiple comparisons). **H** Schematic of the adeno-associated virus (AAV) (upper) and the experimental schedules (lower) after AAV injection into the hippocampus of control and APP/PS1 mice. **I**, **J** Representative images (I) showing the expression of AAV (green) and co-stained NeuN in the neurons of mouse hippocampus (scale bar, 50 μm), and relative expression of Lamr1-ps1 (J) in the hippocampus of mice after being injected with AAV-Lamr-ps1 or AAV-Con. Data are the mean ± SEM (*n* = 3 per group, ***P <0.001, *t =* 12.1, unpaired two-tailed *t*-test). **K**, **L** Cell lysates from the hippocampus of 6-month-old APP/PS1 mice prepared 35 days after AAV-Lamr-ps1 or control injection and blotted with anti-APP, Bace1, Lamr1, and GAPDH, as indicated (K), and the relative protein expressions of APP, Bace1, and Lamr1 (L). Data are the mean ± SEM (*n* = 8 per group, **P = 0.0011, *t =* 4.085 for Bace1 and **P = 0.0031, *t =* 3.569 for Lamr1 in the AAV-Lamr1-ps1-treated group compared to the control group, unpaired two-tailed *t*-test).

We next examined the expression of APP, and the beta-site of APP-cleaving enzyme 1 (Bace1) following the overexpression or knockdown experiment. Given that Lamr1-ps1 is a pseudogene of Lamr1, we also measured Lamr1 protein, which has been reported to be involved in the generation and maturation of APP [18, 23], in a subsequent validation process after the expression of Lamr1-ps1 changed. Total RNA extracted from N2a cells transfected with Lamr1-ps1 plasmid was used to assess the alterations of Lamr1-ps1, Bace1, Lamr1, and APP at the RNA level. The results confirmed that only Bace1 increased in the presence of high Lamr1-ps1 expression (Fig. 3A, B, *P* <0.001). This result was corroborated by the WB assay from the same treated N2a cell lysates (Fig. 3C, D, *P* <0.001). Next, we examined the effect of Lamr1-ps1 knockdown in cells. Since the expression of lncRNA has tissue and cell specificity, and low expression of lncRNA in cells is unfavorable for knockdown, we compared the expression levels of Lamr1-ps1 in these two neural-derived cells (N2a and HT22) and BV2 (a microglial cell line, as a control). The amplification curves and normalized expression suggested that Lamr1-ps1 expression in N2a could be used in subsequent experiments (Fig. S3A, B). Fig. 3E shows the efficiency of knocking down Lamr1-ps1 at three different binding sites. The protein quantification results confirmed that the expression of Bace1 was reduced with decreasing Lamr1-ps1 (Fig. 3F, G).

We next attempted to overexpress Lamr1-ps1 in the hippocampus of WT mice, but surprisingly there was no change in Bace1 expression (Fig. S3C, D), which we thought may be related to the normal regulation of the organismal environment. This led us to speculate whether Lamr-ps1 plays a role in the pathological conditions of AD. Then, we used the 5- and 11-month-old APP/PS1 mice injected with AAV-Lamr1-ps1 or control virus according to the experimental procedures (Fig. 3H). One month later, Lamr1-ps1 virus expression was verified by co-staining brain sections with NeuN and qPCR assay (Fig. 3I, J, *P* <0.001). WB assays indicated that the protein levels of Bace1 and Lamr1 in 6-month-old APP/PS1 mice injected with AAV-Lamr1-ps1 increased compared to control (Fig. 3K, L, *P* <0.001, and *P* = 0.0139, respectively). The same increased alteration of Bace1 was also seen in the 12-month-old group (Fig. S3E, F, *P* <0.001). These results showed that Bace1 is up-regulated by the overexpression of Lamr1-ps1. Taken together, these results demonstrated that Lamr1-ps1 enhances the expression of Bace1.

### Lamr1-ps1 Aggravates Early Learning Memory Deficits in APP/PS1 mice

**Fig. 4:**
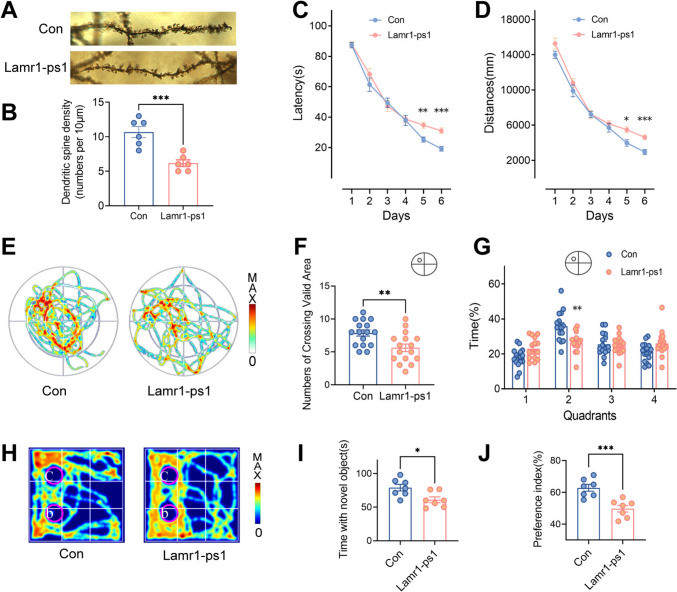
Lamr1-ps1 accelerates the impairment of spatial learning and memory in APP/PS1 mice. **A** Representative images of Golgi staining in the hippocampus (600×) of AD mice after injection of AAV-Lamr1-ps1 or control virus. **B** Numbers of dendritic spines in the hippocampus of each group (*n* = 6 dendrites per group, ***P <0.001, *t =* 4.818, unpaired two-tailed *t*-test). **C**, **D** The MWM test is applied to examine spatial learning and memory in 6-month-old APP/PS1 mice 28 days after AAV-Lamr-ps1 or AAV-Con injection. The latency (C) and the swimming distance (D) to reach a hidden platform in the hidden platform version of the water maze for 6 consecutive days. Data are the mean ± SEM (*n* = 16 per group for latency in the Lamr1-ps1 group compared to control, *F* = 4.498, **P = 0.0078 and ***P <0.001 at days 5 and 6, respectively; for distance, *F* = 11.32, *P = 0.0305 and ***P <0.001 in the Lamr1-ps1 group compared to the control at days 5 and 6, respectively. Two-way ANOVA followed by Bonferroni multiple comparisons). **E** Representative images of motion tracking plotted from probe tests of 6-month-old APP/PS1 mice with AAV-Lamr-ps1 or AAV-Con injection. **F** The number of crossings of effective regions during the probe trial. Data are shown as the mean ± SEM (*n* = 16 mice per group, **P = 0.0049, *t* = 3.04, unpaired two-tailed *t*-test). **G** The percentage of time spent searching for a hidden platform in the target quadrant during the probe trial. Data are shown as the mean ± SEM (*n* = 16 mice per group, *F* = 7.319, **P = 0.0035 in quadrant 2, two-way ANOVA followed by Bonferroni multiple comparisons). **H** Representative images of novel object recognition test from APP/PS1 mice after AAV-Lamr-ps1 or AAV-Con injection. The novel object is placed in position C, where object A the same as B was located in the previous adaptation phase. **I**, **J** The time mice spend with novel object C (I) and the preference index calculated by the time with the object using the formula c/(b+c) × 100% (J). Data are shown as the mean ± SEM (*n* = 7 per group, *P = 0.0174, *t* = 2.757 for time with novel object, and ***P <0.001, *t* = 4.381 for the preference index in the Lamr1-ps1-treated group compared to control, unpaired two-tailed *t*-test).

The subsequent investigation sought to ascertain the impact of Lamr1-ps1 overexpression on neural function. By Golgi staining, we clarified the reduction of dendritic spines upon Lamr1-ps1 overexpression (Fig. 4A, B, *P* <0.001). To assess the spatial learning and memory of APP/PS1 mice infected with AAV-Lamr1-ps1, the MWM test was applied in the 6-month-old group injected with AAV or control. In the spatial learning trial, the mice injected with AAV-Lamr1-ps1 showed substantial differences in both swim distance and latency compared to the control treatment group at the last training session (Fig. 4C, D, *P* <0.001, and *P* <0.001, respectively). From the spatial probe test, we graphically depicted the representative motion tracking hotspots and quantified the time spent in each quadrant of the two groups (Fig. 4E). The number of crossings of the valid area around the hidden target platform and the time spent in the target quadrant were analyzed. The results showed that mice in the treatment group had fewer crossings of the hidden platform (Fig. 4F, *P* = 0.0049) and spent less time in the target 2^nd^ quadrant (Fig. 4G, P = 0.0035) than the control group. The additional novel object recognition test was used to test memory. As shown in Fig. 4H, the old object B and the novel object C were placed in a box and the motion-tracking hotspots were recorded. The results demonstrated that mice in the treated group exhibited a significantly reduced duration (Fig. 4I, *P* = 0.0174) and preference index (Fig. 4I, J, *P* <0.001) for the exploration of novel objects in comparison to those in the control group. These results suggested that overexpression of Lamr1-ps1 aggravates the early spatial learning memory deficits in APP/PS1 mice.

At the end of the behavioral experiments, mice were sacrificed for the examination of protein alteration and Aβ_1-42_ deposits. The results of immunohistochemistry suggested that Aβ plaques were slightly increased in Lamr1-ps1-treated 6-month-old APP/PS1 mice (Fig. S4A). The statistics revealed a significant increase in the number of Aβ plaques and the relative area of Aβ staining following the Lamr1-ps1 intervention (Fig. S4B, C, *P* = 0.0264 and *P* = 0.0084, respectively). The ELISA assay results also showed that the Aβ_1-42_ level was increased in these mice (Fig. S4D, *P* = 0.0018). Subsequently, we examined the alterations of some neural-related receptors and synapse-associated proteins and the result showed that the expression of N-methyl-D-aspartate receptor type 2B was reduced in response to high Lamr1-ps1 expression in both N2a and AD mice (Fig. S5).

### Lamr1-ps1 Regulates the Expression of Bace1 *via* miR-29c-3p

**Fig. 5:**
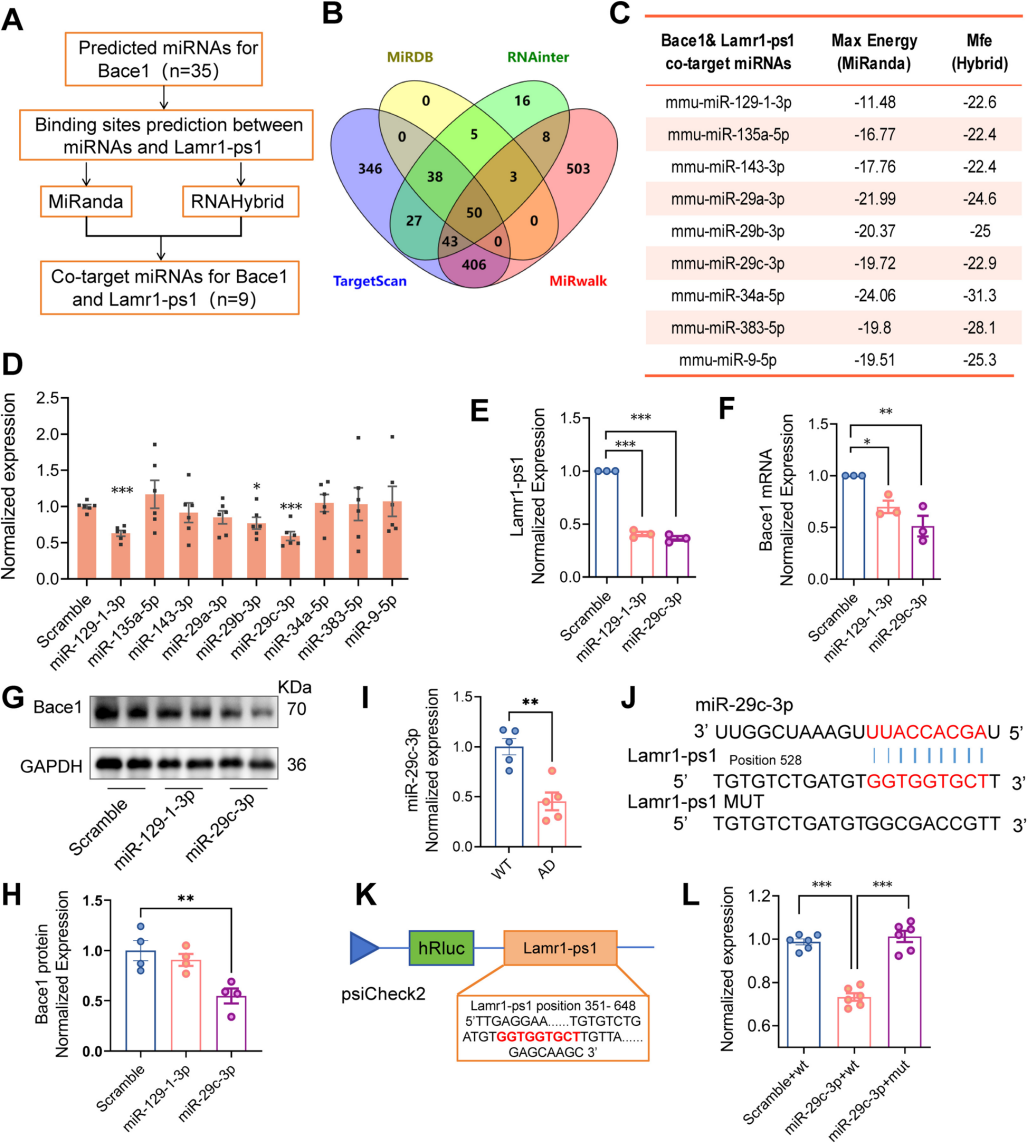
Lamr1-ps1 regulates the expression of Bace1 *via* miR-29c-3p. **A** Prediction flow chart for common miRNA targets of Lamr1-ps1 and Bace1. **B** Venn diagram of the predicted miRNAs targets of Bace1. **C** Interaction scores of candidate miRNAs with Lamr1-ps1 predicted by MiRanda and RNAHybrid algorithms. **D** Normalized expression of candidate miRNAs in N2a cells with Lamr1-ps1 over-expression. Data are shown as the mean ± SEM (*n* = 6 per group, ***P <0.001, *t* = 7.803 for miR-129-1-3p; *P = 0.0226, *t* = 2.692 for miR-29b-3p; ***P <0.001, *t* = 6.048 for miR-29c-3p, compared to the scrambled control group, unpaired two-tailed *t*-test). **E** Normalized expression of Lamr1-ps1 in N2a cells 48 h after treatment with miR-129-1-3p, miR-29c-3p mimics, or scrambled. Data are the mean ± SEM (*n* = 3 per group, *F* = 456.7, ***P <0.001 in the miR-129-1-3p and miR-29c-3p groups compared to scrambled control, one-way ANOVA followed by Bonferroni multiple comparisons). **F-H** Bace1 is regulated by miR-29c-3p, but not miR-129-1-3p. The qPCR validation of Bace1 in N2a at 48 h after treatment with miR-129-1-3p, miR-29c-3p mimics or scrambled (F). The cell lysates from N2a cells at 48 h after treatment with miR-129-1-3p, miR-29c-3p mimics, or scrambled prepared and blotted with anti-Bace1 and GAPDH, as indicated (G). And the relative protein expression of Bace1 (H). Data are the mean ± SEM (*n* = 3 per group for mRNA expression detection, *F* = 13.35, *P = 0.034 and **P = 0.004 for the miR-129-1-3p and miR-29c-3p groups compared to the scrambled control, respectively; *n* = 4 per group for protein expression, *F* = 8.947, **P = 0.006 for the miR-29c-3p group compared to scrambled control, one-way ANOVA followed by Bonferroni multiple comparisons). **I** The expression of miR-29c-3p in APP/PS1 and WT mice. Data are shown as the mean ± SEM (*n* = 5 per group, **P = 0.0018, *t* = 4.562, unpaired two-tailed *t*-test). **J** Schematic of the miR-29c-3p binding region with Lamr1-ps1, and the mutant sequence of the binding region for luciferase analysis. **K** Schematic of plasmid construction used for the luciferase reporter assay. The WT fragment from Lamr1-ps1 at 351-648 bp or the mutant fragment is cloned into the 3’UTR of *Renilla* luciferase on the psiCheck2 plasmid. **L** WT or mutant (MUT) of the Lamr1-ps1 binding sequence in the psi-CHECK-2 vector is co-transfected into HEK293T cells with miR-29c-3p mimic or scrambled control. Luciferase activity was determined at 48 h after transfection. Data are the mean ± SEM (*n* = 6 per group, *F* = 61.28, ***P <0.001 in the miR-29-3p+WT group compared to the scrambled control or miR-29c-3p+MUT group, one-way ANOVA followed by Bonferroni multiple comparisons).

Given the positive regulatory relationship of Lamr1-ps1 with Bace1, we considered the possibility of the presence of a ceRNA mechanism. Through the steps shown in Fig. 5A, we obtained 9 miRNAs that may have binding sites to both Lamr1-ps1 and Bace1. Specifically, candidate miRNA targets of Bace1 were first predicted from the four commonly used databases: TargetScan [24], miRDB [25], miRwalk [26], and RNAinter [27], and plotted using a Wayne diagram (Fig. 5B). The predicted miRNAs present in no less than 3 databases were picked out to analyze the likelihood of combination with Lamr1-ps1 by miRanda (an algorithm integrated in SRplot) [28], and RNAhybrid (a tool for finding the minimum free energy hybridization of a long and a short RNA) [29] for predictive binding sites of these candidate miRNA targets. The details of the combination between miRNAs and Lamr1-ps1 are shown in Fig. 5C. Then, alterations of these candidate miRNAs were examined by qPCR after Lamr1-ps1 was or was not overexpressed in N2a cells. The results suggested that 3 miRNAs, miR-129-1-3p, miR-29c-3p, and miR-29b-3p, were affected by Lamr1-ps1 expression (Fig. 5D, *P* <0.001, *P* <0.001, and *P* = 0.0226, respectively). Subsequently, downregulation of Lamr1-ps1 was verified in cells transfected with a mimic of miR-129-1-3p or miR-29c-3p compared with the control group (Fig. 5E, *P* <0.001 and *P* <0.001, respectively). This suggested that both miR-29c-3p and miR-129-1-3p can cause changes in the expression of Lamr1-ps1. To determine whether these miRNAs affect Bace1, the mRNA level of Bace1 in these cell samples was examined. The results demonstrated that the addition of the miR-29c-3p mimic led to a significant reduction in Bace1 compared to its scrambled control (Fig. 5F, *P* = 0.004). Protein immunoblotting bands and statistics from WB experiments corroborated this finding (Fig. 5G, H, *P* = 0.006). Therefore, we tended to select miR-29c-3p as a possible intermediate molecule between Lamr1-ps1 and Bace1.

Abnormal expression of miR-29c-3p in the AD model has been reported [30, 31]. Here, we examined its expression in AD and WT mice (Fig. 5I, *P* = 0.0018), and the results showed a downregulation of miR-29c-3p in AD mice as previously reported. Next, a dual-luciferase reporter assay was applied to check the binding site between Lamr1-ps1 and miR-29c-3p. Their binding site and mutation control sequence are shown in Fig. 5J and the sequence of ~300 bp which contains the binding site in the Lamr1-ps1 gene was cloned in the 3’UTR of *Renilla* luciferase on the dual-luciferase plasmid psiCheck2 (Fig. 5K). The data showed that miR-29c-3p co-transfected with a plasmid containing the WT sequence of Lamr1-ps1 notably downregulated the luciferase activity compared to the scrambled mimic group or with the mutant sequence (Fig. 5L, *P* <0.001 and *P* <0.001, respectively). Thus, we verified that miR-29c-3p is a downstream target of Lamr1-ps1.

### Therapeutic Amelioration of Learning Memory Deficits in APP/PS1 Mice *via* miR-29c-3p Augmentation

**Fig. 6:**
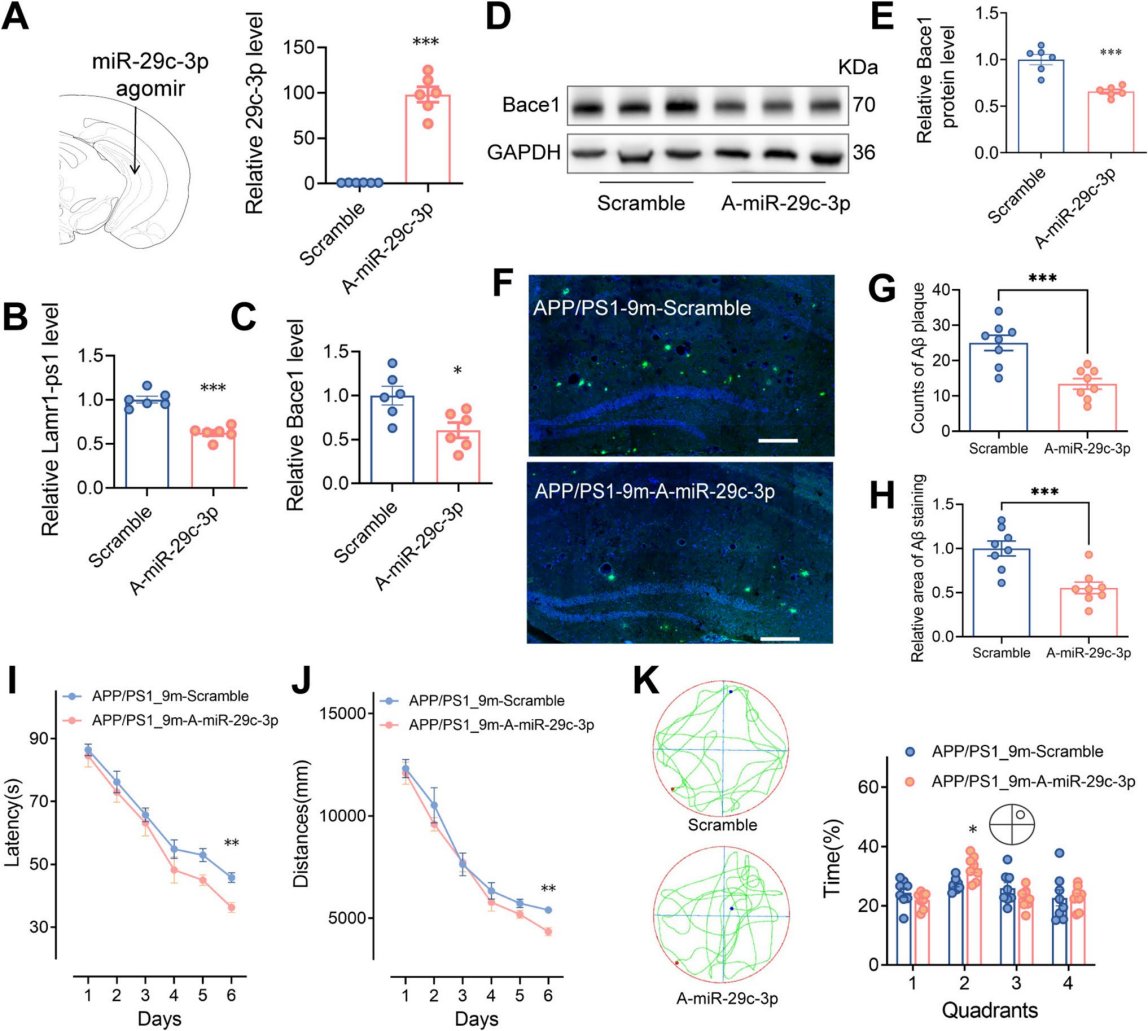
miR-29c-3p ameliorates the cognitive deficits of spatial learning and memory in APP/PS1 mice. **A-C** miR-29c-3p agomir (A-miR-29c-3p) solution or scrambled control is released in the mouse hippocampus (A, upper), and the relative RNA expression of miR-29c-3p (A, lower), Lamr1-ps1 (B) and Bace1 (C) in the hippocampus of APP/PS1 mice 3 weeks after treatment with miR-29c-3p agomir or control. Data are shown as the mean ± SEM (*n* = 6 per group, ***P <0.001, *t* = 11.46 for the expression of miR-29c-3p, ***P <0.001, *t* = 7.690 for the expression of Lamr1-ps1, and *P = 0.016, *t* = 2.872 for the expression of Bace1 in the miR-29c agomir-treated group compared to control, unpaired two-tailed *t*-test). **D**, **E** Expression of Bace1 at the protein level in the hippocampus of APP/PS1 mice treated with miR-29c-3p agomir or control. The hippocampus cell lysates are blotted with anti-Bace1 and GAPDH, as indicated (D) as well as the relative protein expression of Bace1 (E). Data are shown as the mean ± SEM (*n* = 6 per group, ***P <0.001, *t* = 5.724, unpaired two-tailed *t*-test). **F-H** Representative images (F) show the labeling of anti-beta-amyloid in the hippocampus from 9-month-old APP/PS1 mice with miR-29c-3p agomir or control injection (scale bars, 200 μm). The statistical data of the counts (G) and area (H) of Aβ staining plaques. Data are shown as the mean ± SEM (*n* = 8 per group, ***P <0.001, *t* = 4.395; and ***P <0.001, *t* = 4.187, respectively, unpaired two-tailed *t*-test). **I**, **J** The MWM test for spatial learning and memory in 9-month-old APP/PS1 mice 21 days after miR-29c-3p agomir or control injection. The latency (I) and the swimming distance (J) to reach a hidden platform during six consecutive days. Data are the mean ± SEM (*n* = 8 per group, *F* = 10.19 for latency, **P = 0.0045 at day 6 in the A-miR-29c group *vs* control; *F* = 2.943 for distance, **P = 0.0044 at day 6 in the A-miR-29c group *vs* control. Two-way ANOVA followed by Bonferroni multiple comparisons). **K** Representative images of motion-tracking in probe tests for each group (left) and the percentage time spent searching for a hidden platform in the target quadrant during the probe trial (right). Data are shown as the mean ± SEM (*n* = 8 mice per group, *F*= 2.308, *P = 0.025 in quadrant 2, two-way ANOVA followed by Bonferroni multiple comparisons).

Several prior studies have demonstrated that miR-29c-3p regulates the expression of Bace1 [30, 31], but *in vivo* studies, especially in AD model mice, are scarce. Therefore, we next injected miR-29c agomir and control into the hippocampus of 9-month-old APP/PS1 mice that had cognitive impairment, to determine whether the Bace1 level could be modulated and whether the cognitive impairment could be ameliorated (Fig. 6A). The hippocampal tissue was taken for assessment three weeks after miR-29c-3p agomir intervention and the statistics suggested that the RNA level of Lamr1-ps1 in the agomir-treated group decreased compared to control, as well as Bace1 (Fig. 6B, C, *P* <0.001 and *P* = 0.016, respectively). WB experiments confirmed the conclusion that Bace1 is down-regulated in the miR-29c-3p treatment group (Fig. 6D, E, *P* <0.001). These findings show that both Lamr1-ps1 and Bace1 can adsorb miR-29c-3p in the form of a molecular sponge. Furthermore, we assessed the Aβ deposition in the hippocampus of both groups by immunofluorescence staining. The results showed a reduction in Aβ deposition following the administration of miR-29c-3p (Fig. 6F) and the statistics revealed a significant decrease in the number of Aβ plaques and the relative area of Aβ staining following the miR-29c-3p agomir intervention (Fig. 6G, H, *P* <0.001 and *P* <0.001, respectively).

We then evaluated alterations in spatial learning and memory in the two groups using the MWM test. After training, there were substantial discrepancies in swimming distance and latency between the miR-29c agomir-treated group and its counterparts (Fig. 6I, J, *P* = 0.0045 and *P* = 0.0044, respectively). After the last spatial probe test, we depicted the representative motion-tracking hotspots and quantified the time spent in each quadrant of these two groups. The statistics showed an increase in the proportion of mice exploring the target quadrant in the miR-29c treatment group (Fig. 6K, *P* = 0.025).

## Discussion

AD is a neurodegenerative disease with a latent onset and a long latency period, during which Aβ deposition and NFTs, the “early events”, occur long before the clinical symptoms of memory loss [4]. The occurrence of these pathological symptoms is largely due to an imbalance in protein expression, and the aberration of lncRNAs may represent one of the initial events in this imbalance. Therefore, it is necessary to explore changes in the expression profile of lncRNAs in the early stage of AD. In the present study, we have evaluated the abnormal expression of lncRNA throughout the course of AD by using lncRNA expression profile data of 6- and 12-month-old APP/PS1 mice. We have uncovered that the lncRNA Lamr1-ps1, a pseudogene of the laminin receptor, aggravates the early spatial learning/memory deficits in APP/PS1 mice. Based on bioinformatics prediction and experimental validation, we have concluded that Lamr1-ps1 might play these roles *via* the miR-29c/bace1 pathway. Experimentally increasing the levels of miR-29c-3p in the hippocampus partially alleviated the spatial learning and memory deficits in AD mice. This finding demonstrates that Lamr1-ps1, through the miR-29c/bace1 pathway, accelerates the progression of early AD and leads to learning and memory impairment.

Pseudogenes are fragments of DNA that closely resemble the sequence of some genes with coding functions, yet do not code proteins due to mutations in the open reading frame, and some pseudogenes have been classified as biologically similar to lncRNAs [17]. To our knowledge, the present study is one of only a few articles on the mechanism of pseudogenes in AD. Lamr1-ps1 was found here to be abnormally expressed in the early stages of APP/PS1 mice. A finding in 1997 reported that nuclear-localized mtDNA pseudogenes show heteroplasmy in AD patients and normal individuals [32]. Few studies on the molecular mechanism of pseudogenes in AD have been reported. A recent study found that the pseudogene ACTBP2 (actin beta pseudogene 2) increases blood-brain barrier permeability by regulating the transcription of KHDRBS2 in an Aβ_1-42_ microenvironment [33]. A study on Parkinson’s disease described a ceRNA network of pseudogene GBAP1 (also known as GBA1LP, glucosylceramidase beta 1-like) and GBA by adsorbing miR-22 [34]. In the present study, we revealed that Lamr1-ps1 aggravates early spatial learning/memory deficits in 6-month-old APP/PS1 mice. This provides a new perspective on the role of pseudogenes in AD.

Lamr1 is a 37 KDa/67 KDa laminin receptor located within the cholesterol-rich lipid raft domains of the plasma membrane [35]. Several lines of evidence have identified a strict link between APP, Aβ, and Lamr1 [18, 23, 36, 37]. The potential function of Lamr1-ps1 we initially hypothesized might be achieved by targeting Lamr1, as it is a corresponding coding gene of Lamr1-ps1. However, no change occurred in Lamr1 expression resulting from an increase of Lamr-ps1 in normal model cells or animals (Figs 3B-D, S3C, D), but this anticipated rise did occur in AD model mice. The uncertainty regarding the result of the expression of Lamr1 was inconsistent with the envisaged conclusion. It was reassuring that we found Bace1 was stably altered by the overexpression of Lamr1-ps1 in both cells and AD mice (Fig. 3). Therefore, we postponed further exploration of the mechanism of Lamr1 and instead concentrated on Bace1.

The miRNA-mediated ceRNA network was our initial direction for investigating the potential mechanism of action of Lamr1-ps1 on Bace1. Our experimental evidence revealed that miR-29c-3p was adsorbed by both Lamr1 and Bace1, and the overexpression of miR-29c-3p in the hippocampus of AD mice ameliorated spatial learning/memory deficits. According to a previous report, miR-29c-3p attenuates Aβ-induced neurotoxicity by targeting BACE1 in a cellular AD model of PC12 [30], and such a neuroprotective effect of miR-29c has also been reported in SAMP8 mice [31]. It is worth pointing out that our results demonstrated that miR-29c-3p has an ameliorating effect on cognitive impairment in APP/PS1 mice. Furthermore, miR-129-1-3p was identified as another potential candidate miRNA. The luciferase assay results indicated an association between miR-129-1-3p and Lamr1 (Fig. S6). However, no changes in Bace1 were found upon miR-129 expression, suggesting the presence of other molecular targets downstream of the Lamr1-ps1/miR-129-1-3p pathway to be explored. Therefore, the exploration of Lamr1-ps1’s mechanism in AD remains incomplete. Further investigation into the targets of the relevant pathway of Lamr1-ps1/miR-129 will enhance our understanding of how Lamr1-ps1 works in AD.

In this study, we explored the alteration of spatial memory in mice after Lamr1-ps1 overexpression using the water maze behavioral test. We concluded that Lamr1-ps1 can accelerate this cognitive decline in AD model mice, and this was similarly confirmed by the results of novel object recognition experiments (Fig. 4). Although we found a reduction in the dendritic spines on mice hippocampal neurons, the specific mechanism remains to be further explored. It is worth mentioning that we did not find a significant cognitive decline in 12-month-old AD mice after Lamr1-ps1 innervation when compared with control AD mice (Fig. S7), which may be related to the fact that they are severely cognitively impaired. These results may suggest that Lamr1-ps1 can aggravate cognitive impairment in early AD mice.

## Conclusion

In summary, our findings provide a new theoretical basis for the involvement of the pseudogene Lamr1-ps1 in AD. Through a combination of bioinformatics prediction and experimental validation, we confirmed a link between the pseudogene Lamr1-ps1, Bace1, and miR-29c-3p. The result that augmenting miR-29c-3p levels in the hippocampus ameliorated spatial learning and memory deficits consolidated the theoretical basis for miR-29c as a potential therapeutic target for AD.

## Supplementary Information

Below is the link to the electronic supplementary material.

Supplementary file1 (PDF 1161 kb)

## Data Availability

The data that support the findings of this study are available from the corresponding author upon reasonable request.
