# Supplementary material for: Pseudogene Lamr1-ps1 Aggravates Early Spatial Learning Memory Deficits in Alzheimer’s Disease Model Mice
Source: Neurosci Bull. 2025 Jan 2;41(4):600–14. doi: 10.1007/s12264-024-01336-6 (PMC11979086; doi:10.1007/s12264-024-01336-6)
Supplement: Supplementary file 1 — Supplementary file1 (PDF 1161 kb) [file 12264_2024_1336_MOESM1_ESM.pdf]

Supplementary Information

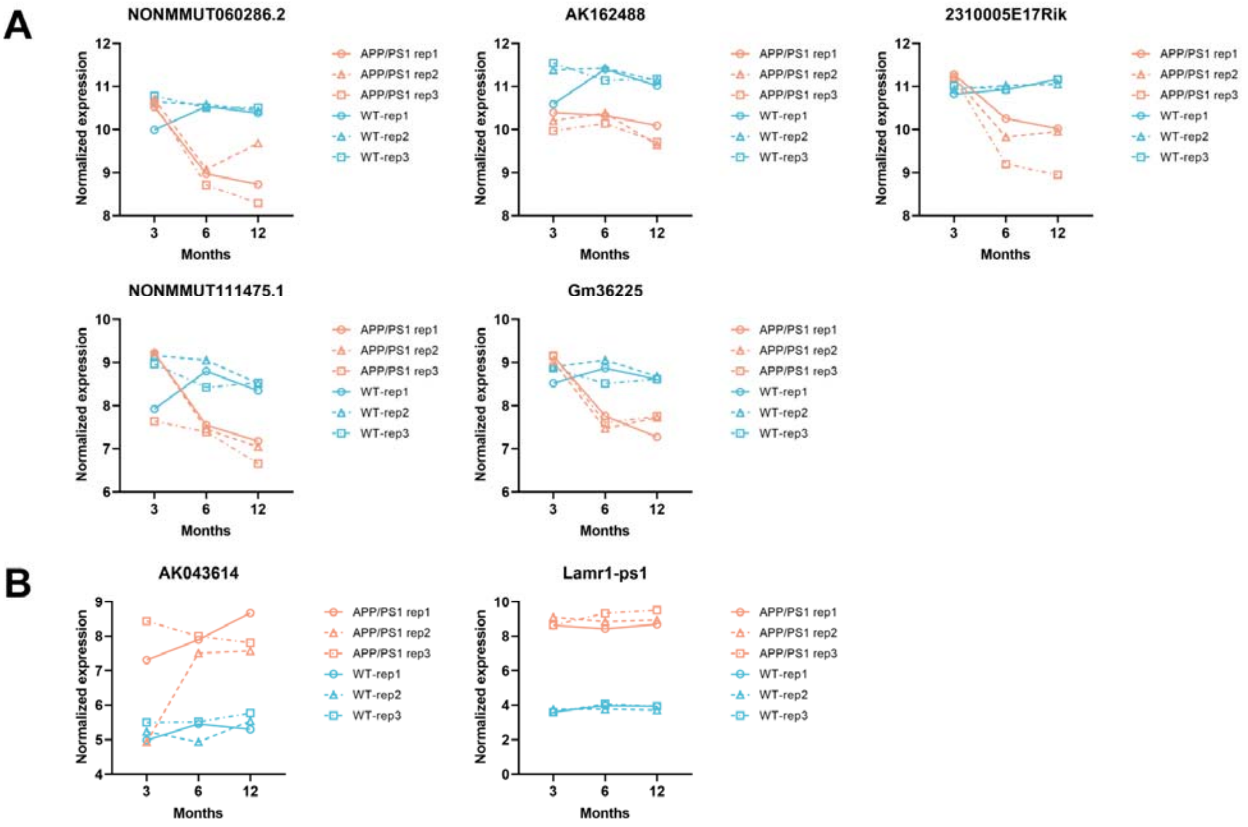

C

	A6M vs. W6M			A12M vs. W12M						
LncRNA Name	*P_value*	FC (abs)	Regulation	*P_*value	FC (abs)	Regulation	Genesymbol	Genebank acc	NONCODEID	ProbeName
2310005E17Rik	0.0178	2.29	down	0.0130	2.80	down	2310005E17Rik	NR_045498	NONMMUT018816.2	A_55_P2240116
AK162488	0.0009	2.06	down	0.0009	2.45	down	/	AK162488	NONMMUT020635.2	CUST_OEV3_068113
Gm36225	0.0026	2.30	down	0.0026	2.07	down	Gm36225	XR_397005	NONMMUT099683.1	CUST_OEV3_066094
NONMMUT060286.2	0.0001	3.08	down	0.0199	2.92	down	/	/	NONMMUT060286.2	CUST_OEV3_054620
NONMMUT111475.1	0.0024	2.45	down	0.0008	2.84	down	/	/	NONMMUT111475.1	CUST_OEV3_021922
LncRNA Name	*P_*value	FC (abs)	Regulation	*P_*value	FC (abs)	Regulation				ProbeName
AK043614	0.0005	5.65	up	0.0023	5.59	up	/	AK043614	NONMMUT038737.2	CUST_OEV3_061801
Lamr1-ps1	0.0001	30.51	up	0.0000	36.31	up	Lamr1-ps1	/	NONMMUT039982.2	CUST_OEV3_061347

Fig. S1 Information and expression profiles of candidate DElncRs. **A**, **B** The expression profiles of the seven DElncRs; the expression curves of five down (A) and two up (B) -regulated DElncRs. The lines for each replicated sample show the changing trend. **C** The detailed name information of these seven candidate DElncRs in official databases, and their expression changes in AD mice.

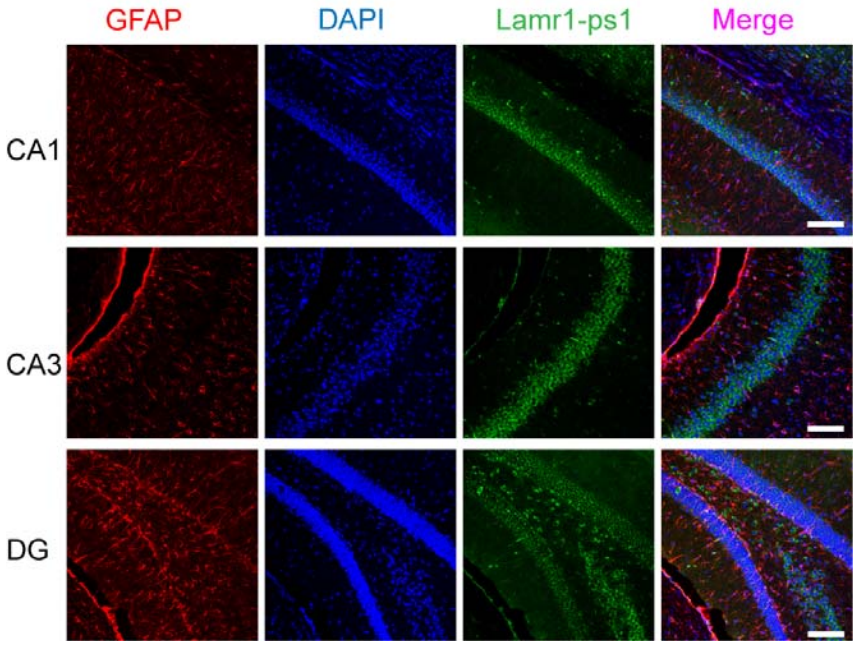

Fig. S2 Representative images showing the expression of Lamr1-ps1 (green) and GFAP (a marker of astrocytes, red) in the hippocampus of APP/PS1 mice. The stained images in CA1, CA3, and the DG are plotted from upper to lower, and the enlarged images show the co-staining of astrocytes and Lamr1-ps1 (scale bars, 100 μm).

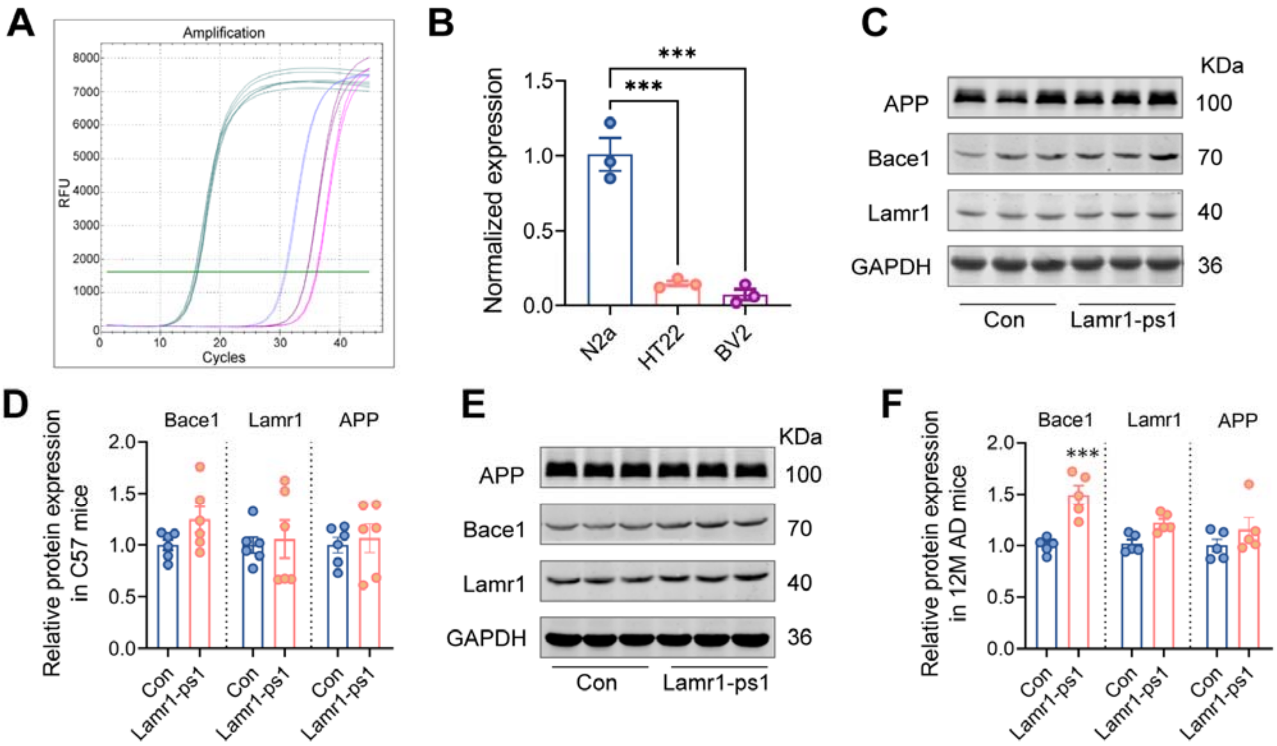

Fig. S3 Lamr1-ps1 is upstream of Bace1. **A** Representative amplification curves of Lamr1-ps1 in three cell samples, starting from left 2 (left 1 is GAPDH of all samples) to right are from N2a, HT22, and BV2. **B** Relative expression of Lamr1-ps1 in the three cell types. Data are the mean ± SEM (*n* =3 per group, Sh-Con *vs* ShRNA3 **P* = 0.021, *F* = 1.58, one-way ANOVA followed by Bonferroni multiple comparisons). **C**, **D** Cell lysates from the hippocampus of C57 mice after AAV injection, blotted with anti-APP, Bace1, Lamr1, and GAPDH, as indicated (C). Relative protein expression of APP, Bace1, and Lamr1 (D). Data are the mean ± SEM (*n* = 6 per group, no significant difference, unpaired two-tailed *t*-test). **E**, **F** Cell lysates from the hippocampus of 12-month-old APP/PS1 mice 35 days after AAV injection, blotted with anti-APP, Bace1, Lamr1, and GAPDH, as indicated (K). Relative protein expression of APP, Bace1. and Lamr1 (L). Data are the mean ± SEM (*n* = 5 per group, *F* = 11.46, ****P* <0.001 and **P* = 0.0139 for Bace1 and Lamr1, respectively, Two-way ANOVA followed by Bonferroni multiple comparisons.

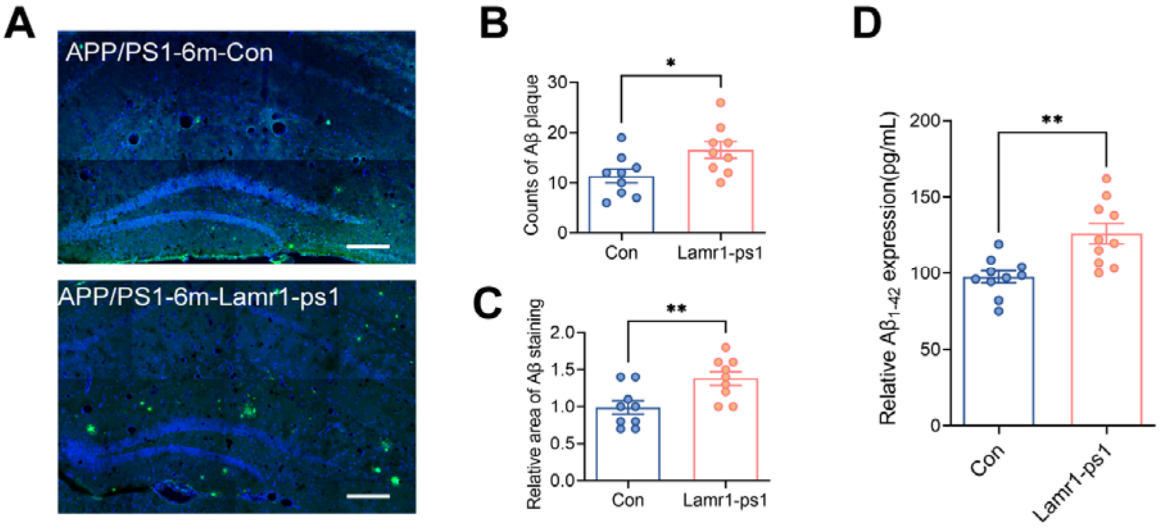

Fig. S4 Lamr1-ps1 increases Aβ deposition in 6-month-old APP/PS1 mice. **A** Representative images showing the labeling of anti-beta-amyloid in the hippocampus of these mice injected with AAV-Lamr1-ps1 or control virus (scale bars, 200 μm). **B**, **C** Statistics of the counts (B) and area (C) of Aβ-staining plaques. Data are shown as the mean ± SEM (*n* = 9 per group, **P* = 0.0264, *t* = 2.446; and ***P* = 0.0084, *t* = 3.007, respectively, unpaired two-tailed *t*-test). **D** The relative expression of $A\beta_{1-42}$ in hippocampus from 6-month-old APP/PS1 mice injected with AAV-Lamr1-ps1 or control homogenized and processed for ELISA determination. Data are the mean ± SEM (*n* = 10 per group, ***P* = 0.0018, *t* = 3.656, unpaired two-tailed *t*-test).

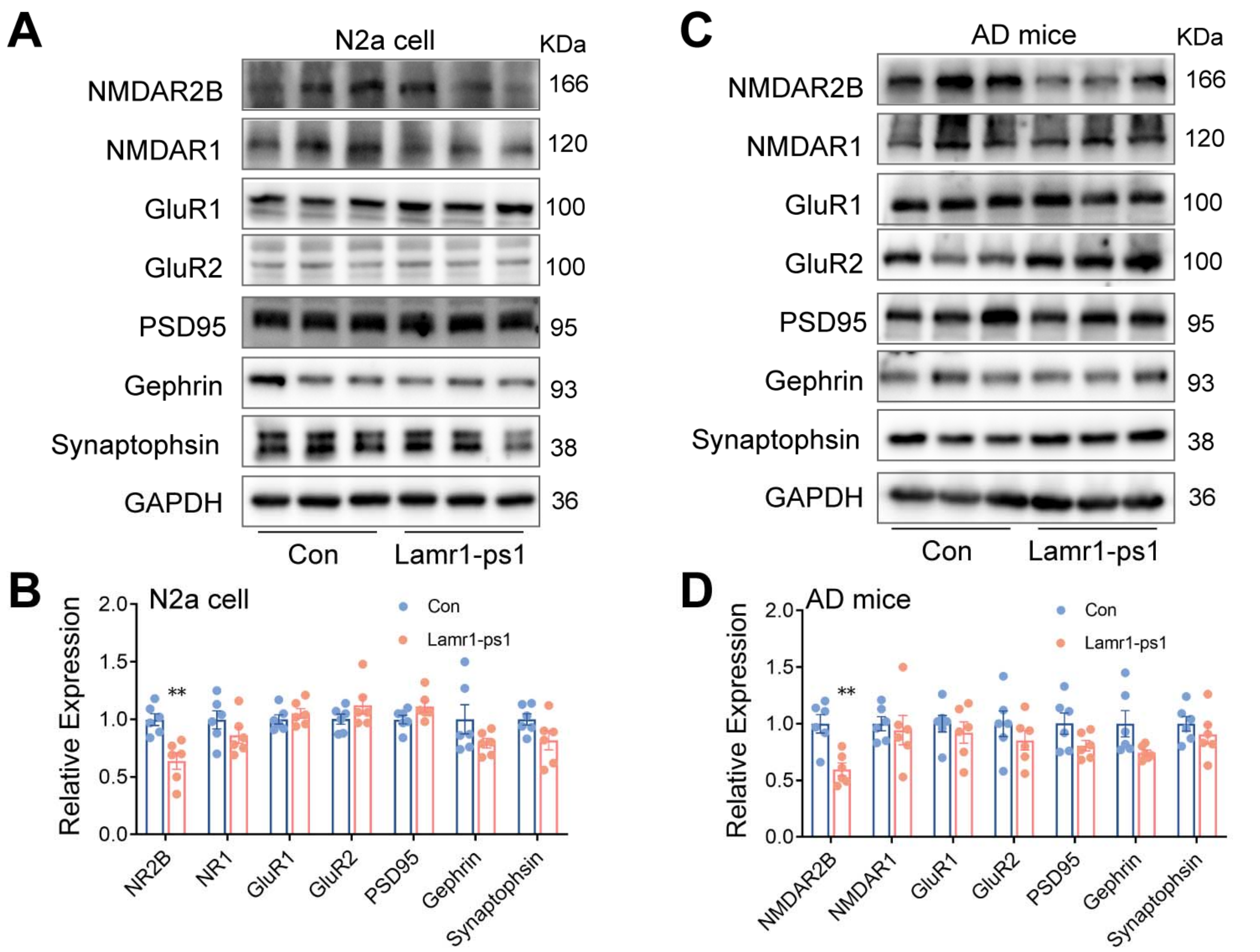

Fig. S5 Lamr1-ps1 causes changes in proteins associated with synaptic functions. **A-D** Lysates from N2a cells (A, B) and the hippocampus of AD mice (C, D) with Lamr1-ps1 overexpression or controls blotted with the indicated antibodies (A, C). Relative protein expression defined by normalizing their respective band intensity to GAPDH (B, D). Data are the mean ± SEM (n = 6 per group, in N2a cells, $^{**}P = 0.0019$, t = 3.84 for NMDAR2B between the Lamr1-ps1-treated group and control; in AD mice, $^{**}P = 0.0089$, t = 3.357 for NMDAR2B, unpaired two-tailed t-test).

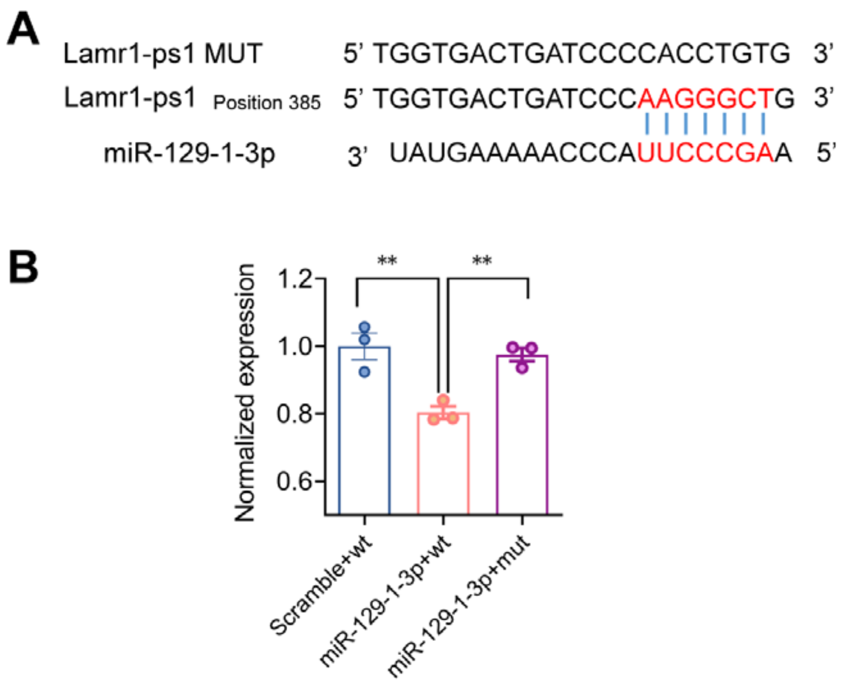

Fig. S6 miR-129-1-3p can be bound by Lamr1-ps1. **A** Schematic showing the miR-129-1-3p binding region with Lamr1-ps1, and the mutant sequence of the binding region for luciferase analysis. **B** The wild-type (WT) or mutant (MUT) Lamr1-ps1 binding sequence in the psi-CHECK-2 vector is co-transfected into HEK293T cells with miR-129-1-3p mimic or scrambled control. The luciferase activity is determined 48 h after transfection. Data are the mean ± SEM (n = 3 per group, F = 14.94, **P = 0.0043 in the miR-129-3p+WT group compared to the scrambled control; and **P = 0.0083 in the miR-129-3p+WT group compared to the miR-29c-3p+MUT group, one-way ANOVA followed by Bonferroni multiple comparisons).

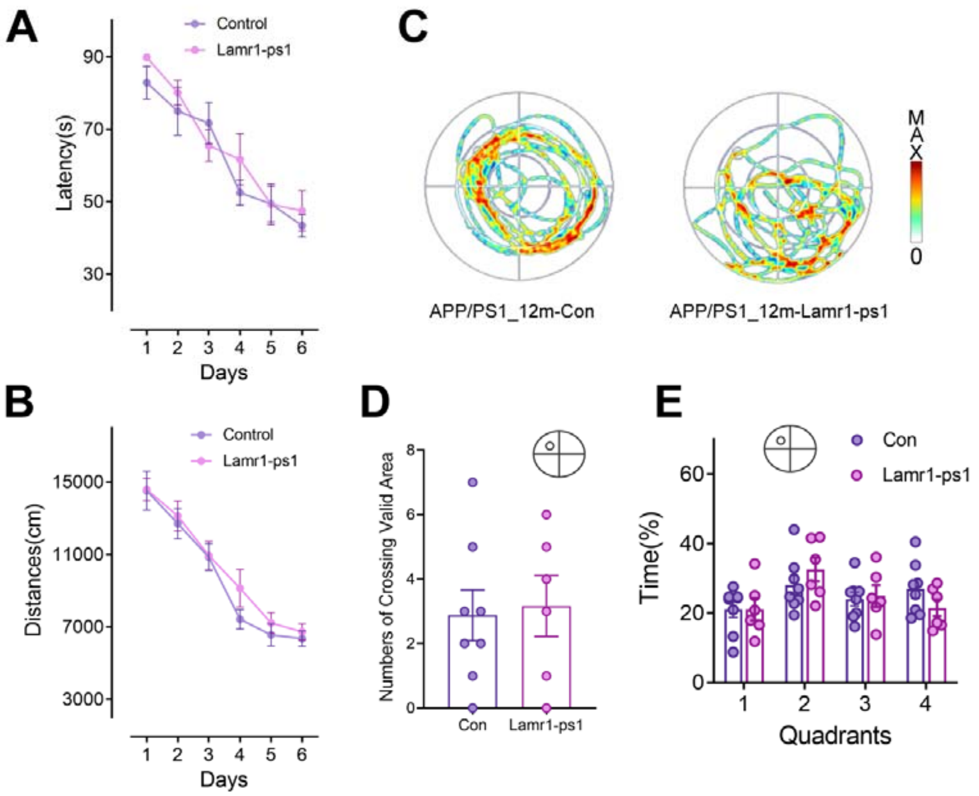

Fig. S7 Effect of Lamr-ps1 overexpression on spatial cognition in 12-month-old APP/PS1 mice. **A**, **B** The MWM test is applied to examine spatial learning and memory in 12-month-old APP/PS1 mice 28 days after AAV-Lamr-ps1 or AAV-Con injection. The latency (A) and the swimming distance (B) to reach the hidden platform during six consecutive days. Data the are mean ± SEM (*n* ≥7 per group, two-way ANOVA). **C** Representative images of motion tracking from probe tests in 12-month-old APP/PS1 mice injected with AAV-Lamr-ps1 or AAV-Con. **D** Numbers of crossings of the effective region during the probe trial. Data are shown as the mean ± SEM (*n* ≥7 per group, unpaired two-tailed *t*-test). **E** Percentage of time spent searching for a hidden platform in the target quadrant during the probe trial. Data are shown as the mean ± SEM (*n* ≥7 mice per group, two-way ANOVA).

Table S1

Primers for qPCR			
Probes	Target ID		Sequence (5'-3')
A_55_P2240116	NONMMUT018816.2	Forward	AACACACAGGAGTTTACGGC
		Reverse	TAAACACCACCACCATGAAC AGT
CUST_OEV3_066094	NONMMUT099683.1	Forward	CGGGAAGAGCTGTGAGCAAA
		Reverse	CGCTGTCAAGAAGGGGACAT
CUST_OEV3_021922	NONMMUT111475.1	Forward	GCACGATGAAGTTCCCGTTTA
		Reverse	TTCTTTCATGTGGTGCTCGC
CUST_OEV3_068113	NONMMUT020635.2	Forward	CGTGTCTACGCATGTACGGA
		Reverse	GAGGTTGGCTGGGGGATTAG
CUST_OEV3_054620	NONMMUT060286.2	Forward	TCTCGATGTCCTGATGGTGG
		Reverse	CCAACCAACTCCTCGCTACA
CUST_OEV3_061801	NONMMUT038737.2	Forward	TGGTGCTCTGAGCCGAATTT
		Reverse	ACAGCAGCTACATGTGGATTT
CUST_OEV3_061347	NONMMUT039982.2	Forward	GCATCTGGTCTGAAGGTG
		Reverse	AAGTGGGAACTGCCAATC
	APP	Forward	TTCGCTGACGGAAACCAAGA
		Reverse	CGTCAACAGGCTCGACTTCA
	Bace1	Forward	AGACCGACGAGGAATCGGA
		Reverse	AGGATGTTGAGCGTCTGTGG
	Lamr1	Forward	CGGCGTTGTTCTTGATTCCC
		Reverse	CCGCAGCAAGGAATTTGAGG
	GAPDH	Forward	TCATCCCAGAGCTGAACG
		Reverse	TCATACTTGGCAGGTTTCTCC

Table S2

ShRNA for Lamr1-ps1	Sequence (5'-3')
ShRNA1	GCCATGGTATCTGCATGATAATTCAAGAGATTAT CATGCAGATACCATGGCTTTTT
ShRNA2	GCACGGTATAAGACACATTCTTTCAAGAGAAGAA TGTGTCTTATACCGTGCTTTTT
ShRNA3	GGAAGAATGATTGGCAGTTCCTTCAAGAGAGGAA CTGCCAATCATTCTTCCTTTTT
